# 

**Published:** 1866-09

**Authors:** 


### CONTENTS FOR SEPTEMBER.

ORIGINAL CONTRIBUTIONS.

Two Cases of Thoracentesis. By J. W. Freer, M. D .......................p. 385

A Case of Menstruation by a Child Five Years of Age. By A. E. Ames, M. D .......... 387

Excision of Four Inches of the Right Humerus subsequent to Excision of the Head and Upper Third—Recovery with a Useful Arm. By A. J. Baxter, M. D .......... 388

A Case of Locomotor Ataxy. By Henry T. Godfrey, M. D .......... 390

Gonorrhœa Caused by Leucorrhœa. By Benjamin Durham, jr., M. D. 391

Two Cases of Poisoning and Two Cases of Surgery. By H. Wanzer, M. D. 394

HOSPITAL REPORTS.

Surgical Clinic. By R. G. Bogue, M. D .......... 398

PROCEEDINGS OF MEDICAL SOCIETIES.

Chicago Medical Society .......... 401

SELECTED ARTICLES.

The Treatment of Certain Functional and Organic Affections of the Nervous System. By Prof. C. E. Brown-Sequard, M. D .......... 405

Early Viability of the Fœtus. By Wm. Kennedy, M. D .......... 414

EDITORIAL.

*Book Notices*—Clinical Lectures by Prof. A. von Graefe, on Amblyopia and Amaurosis, and the Extraction of Cataract, 417—Cholera: Facts and Conclusions as to its Nature, Prevention and Treatment, 426—Shakspeare's Delineations of Insanity, Imbecility and Suicide, 428—Medical Diagnosis .......... 429

County Hospital .......... 429

Memorandum for those desirous of entering the Army Medical Corps.... 430

